# Supplementary material for: Workflow and Strategies for Recruitment and Retention in Longitudinal 3D Craniofacial Imaging Study
Source: Int J Environ Res Public Health. 2019 Nov 12;16(22):4438. doi: 10.3390/ijerph16224438 (PMC6888265; doi:10.3390/ijerph16224438)
Supplement: Supplementary file 1 [file ijerph-16-04438-s001.zip › Table S2.docx]

**Questionnaire for teachers**

| **Questions** | **Please circle it** |
| --- | --- |
| Example: Is the child full of gain at this event? | 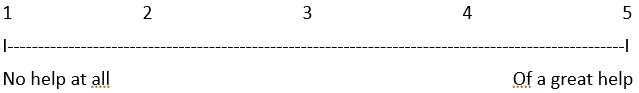 |
| 1. In Pediatric Psychotherapy Station, what do you think of the child's gain and satisfaction? | 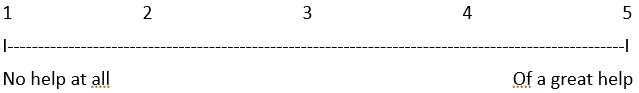 |
| 1. In Pediatric Psychotherapy Station, through the introduction of projection activities, do you know more about the inner mind in children? | 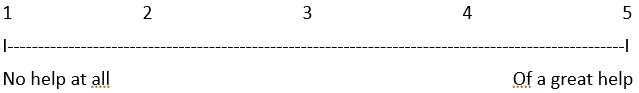 |
| 1. At the Dental Education Station, do you think children can understand the importance of dental care and occlusive concept？ | 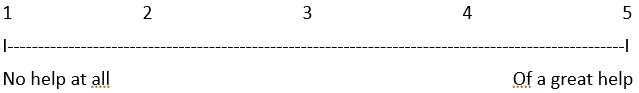 |
| 1. By Pediatric Psychotherapy and Dental Education Stations, do you think it helps children's school daily life？ | 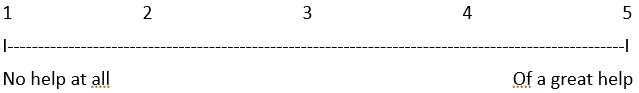 |
| 1. Do you know the significance and contribution of participating in cranial 3D photography? | 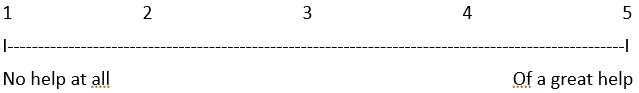 |
| 1. When the activity is summarized, the research staff introduces the meaning and contribution of this activity, do you have a deep understanding？ | 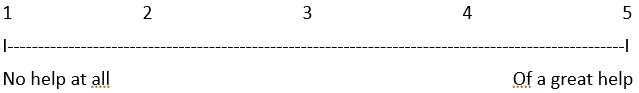 |
| 1. Overall, do you think there is a positive gain for children, parents, and school？ | 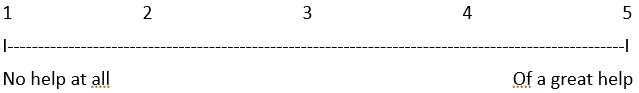 |
| 1. Are you willing to assist in the promotion and participation of the event again next year？ | 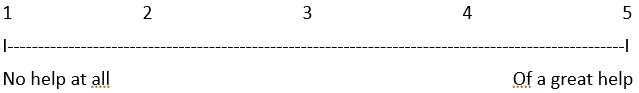 |
