# Supplementary material for: Workflow and Strategies for Recruitment and Retention in Longitudinal 3D Craniofacial Imaging Study
Source: Int J Environ Res Public Health. 2019 Nov 12;16(22):4438. doi: 10.3390/ijerph16224438 (PMC6888265; doi:10.3390/ijerph16224438)
Supplement: Supplementary file 1 [file ijerph-16-04438-s001.zip › Table S1.docx]

**Questionnaire for students**

| **Questions** | **Please circle it** |
| --- | --- |
| Example: Is the child full of gain at this event? | 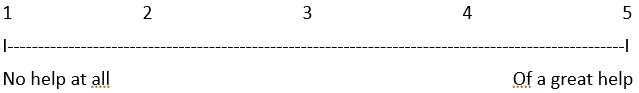 |
| 1. In the Heroes Journey Six-frame comics event, how satisfied are you with the content and methods of the activities led by our staff? | 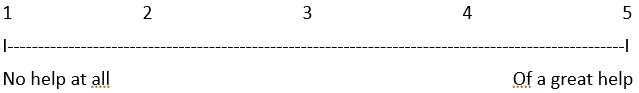 |
| 1. In the Heroes Journey Six-frame comics event, how satisfied are you with yourself after completing the event? | 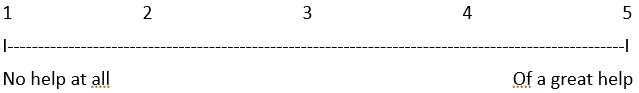 |
| 1. At the brushing station, do you think the knowledge of dental care and occlusion is important? | 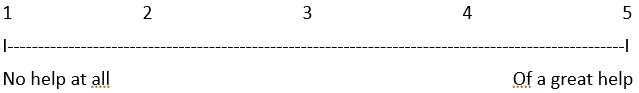 |
| 1. At the brushing station, do you think it is helpful to go back to life? | 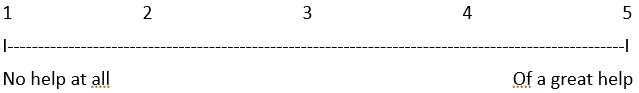 |
| 1. Do you know the significance and contribution of participating in the Cranial 3D capture Camp? | 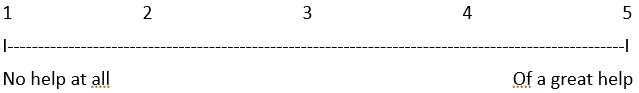 |
| 1. The research staff introduced the meaning and contribution of this activity. Are you clear? | 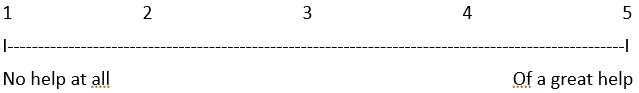 |
| 1. Overall, come to the hospital to participate in the event, how satisfied are you with this event? | 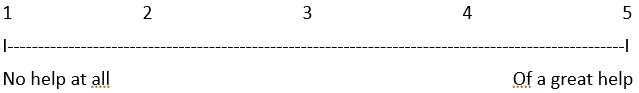 |
| 1. Will you come back to the hospital next year to participate of this activity? | 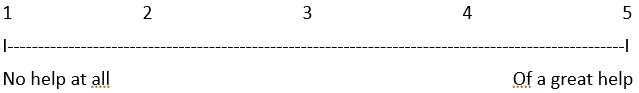 |
